# Patient attitudes and experiences towards exercise during oncological treatment. A qualitative systematic review

**DOI:** 10.1007/s00520-024-08649-2

**Published:** 2024-07-12

**Authors:** Alice Finch, Alex Benham

**Affiliations:** 1 Oncology Therapies Department, St Bartholomew’s Hospital, Barts Health NHS Trust, London, EC1A 7BE UK; 2 School of Allied Health Professions, Keele University, Keele, ST5 5BG UK

**Keywords:** Physical activity, Cancer treatment, Qualitative review, Experiences

## Abstract

**Purpose:**

Exercise and physical activity (PA) during oncological treatment have many benefits. However, PA levels and adherence are often low. This systematic review of qualitative literature aims to explore the experience and the perceived barriers and facilitators to exercise and physical activity during treatment.

**Methods:**

A systematic search of the published literature was carried out in the Embase and Medline databases; full details for the protocol can be found in the Prospero database (CRD42022371206). Studies eligible for inclusion were qualitative and included participants that were either currently undergoing oncological treatment or had finished treatment within the last 6 months. The findings from each study were tabulated and synthesised into analytical themes.

**Results:**

Eighteen full texts from 309 studies met inclusion criteria with a total of 420 participants including both curative and palliative treatment intents. Four overarching themes were generated: (1) Facilitators; (2) Barriers; (3) Experience of PA/exercise and (4) Transforming attitudes. Sub-themes that showed perceptions of PA or exercise during treatment were positive, and seeing personal positive change was highly motivating, especially in a group class setting. Barriers included lack of support or guidance from healthcare professionals (HCPs), environmental challenges and disease burden/fear or worsening symptoms.

**Conclusions:**

Despite having positive perceptions of exercise and PA during oncological treatment, there are significant barriers impacting participation. Lack of support from HCPs and fear of worsening symptoms were significant barriers. Future research should focus on impacting these barriers to ultimately improve PA and exercise levels in those undergoing oncological treatment.

## Introduction

In the UK, one in two people will be diagnosed with cancer in their lifetime, and with 50% of people diagnosed with cancer surviving for 10 years or more [1]. In the USA, over 1.9 million people were diagnosed with cancer in 2023, and there were over 600,000 cancer-related deaths [2]. Although incidences of prostate and stomach cancer decrease globally, the World Cancer Report published in 2020 demonstrates a rise in incidences of breast and colorectal cancer [3]. With cancer impacting so many people globally, it is imperative that support is provided for people to live well with and beyond cancer. The side effects of any oncological treatment can be severe and include breathlessness, fatigue, muscle wastage, weight loss and pain to name a few [4]. The diagnosis itself and associated treatment side effects experienced by people with both curable and incurable cancers have a significant impact on Quality of Life (QOL) [5–9].

Exercise and increased physical activity have many evidenced benefits for patients undergoing cancer treatment [10] such as attenuating for side effects of oncological treatment including improved cardiovascular fitness and strength and reduced fatigue, insomnia and breathlessness [11, 12]. On a cellular level, promising evidence suggests exercise can reduce spread of metastasis and tumour growth [13, 14]. A small RCT found that during neoadjuvant treatment, an exercise intervention group had significantly more tumour regression and downstaging when compared to non-exercise control [15], supporting this promising evidence. Evidence also suggests that exercise may be cardioprotective, attenuating the risk of cardiotoxicity during neoadjuvant treatment [16–18], highlighting the role exercise could play in reducing treatment-related toxicities.

From a surgical perspective, exercise intervention improves pre-operative fitness [19, 20] which in turn reduces postoperative complications and length of stay whilst increasing speed of functional recovery [21–23]. Pre-operative exercise intervention may also be associated with improved disease-free survival in people with colorectal cancer [24] which is supported by a systematic review reporting higher levels of pre and post diagnosis fitness being associated with improved survival outcomes for at least 11 cancer types [25].

Despite this abundance of evidence highlighting the clear benefit of exercise for people undergoing oncological treatment, reported adherence to exercise programmes remains low. One study found only 9% of breast cancer patients adhered to physical activity guidelines (150 min moderate and 75 high intensity exercise per week) [26], and another systematic review found that as few as 44% of advanced cancer patients adhered to prescribed exercise programmes [27]. Furthermore, a 2023 study demonstrated 57.6% of people living with breast, prostate and colorectal cancer did not meet the weekly physical activity and exercise guidelines (*N* = 5385) [28]. Finally, a 2023 systematic review reports that most people with cancer undergoing chemotherapy do not meet physical activity guidelines [29].

This systematic review aims to explore and understand the experience and the perceived barriers and facilitators to exercise and physical activity in those undergoing oncological treatment. This will support future patient-centred advice, design and implementation of PA or exercise interventions by HCPs.

## Methods

This qualitative systematic review was prospectively registered in the Prospective Register of Systematic reviews (PROSPERO ID CRD42022371206).

A comprehensive literature search was conducted in the Medline and Embase databases from inception to October 2022. The search strategy was designed and carried out around the keywords of Cancer or Oncological Treatment, Physical activity or Exercise and studies with Qualitative Methodology. Please see appendix one for full search used (Appendix 1).

### Study selection

Studies were included that reported the experiences or views towards exercise or physical activity whilst undergoing oncological treatment for any form of cancer diagnosis; both solid (e.g. lung or gynaecological tumours) and blood cancers (e.g. leukaemia or myeloma). An exercise intervention was not necessarily required as long as the study was exploring patient’s thoughts and experiences towards PA/exercise. It was felt that understanding and exploring views of those who may not have had an exercise intervention was essential to gain better understanding of perceived positive or negative views and barriers towards exercising independently during oncological treatment. For this review, we considered exercise as any structured supervised/unsupervised/virtual or tele-rehab/advice on exercise or other physical activity given as an intervention. We only included studies where exercise or physical activity was the main phenomenon of interest.

The included studies used qualitative methods to collect in-depth, open information such as one-to-one or semi-structured interviews or focus groups. Studies using only questionnaires or surveys were excluded. No limits were placed on the methodological approach of any of the identified studies or study quality.

### Participants

We included information from all people who were undergoing oncological treatment due to their cancer diagnosis with no constraints on age. We included studies which collected data pre-surgery but only if the participants were on neoadjuvant treatment (pre-surgical cancer treatment, e.g. chemotherapy/radiotherapy/immunotherapy). All countries and all settings were included (primary, secondary or community care, inpatient or outpatient), and no restrictions were placed on date, although language limits were restricted to papers in English only.

### Quality appraisal

The Joanna Briggs Institute (JBI) Critical appraisal checklist for qualitative research [30] was used by both researchers (AF and AB) to independently assess the quality appraisal of the studies. These were reviewed and discussed after to reach a consensus and resolve any disagreement. Studies were not excluded based on the quality appraisal, but the scores were used as part of the judgement and discussion on the basis of the results.

### Procedure

The searches were carried out and imported into the Rayyan web application [31] to identify and remove duplicates and identify appropriate articles. Two reviewers (AF and AB) independently screened all titles and abstracts using the review’s inclusion/exclusion criteria, first by title, then abstract and finally full text. This process was carried out blind, and any disagreements discussed at the end of each stage between the two reviewers.

Once the included papers had been identified, a standardised extraction form was used to record the author, country and year published, the sampling approach, participant characteristics, data collection method, key themes identified, and the related data extracted from them and general appraisal of the study quality. The direct participant quotes made by the participants were also extracted.

### Data synthesis

The descriptive themes and supporting quotes were extracted from the identified primary studies separated by both authors and synthesised using the approach outlined by Thomas and Harden [32]. These initial themes were tabulated and synthesised through author discussion to identify analytical themes. Identified themes and their accompanying quotes were coded individually by two researchers to identify descriptive themes from the included studies. These were then analysed and compared by the researchers individually to identify common themes within the data, and synthesised to identify analytical themes and sub-themes.

## Results

### Search outcomes

**Fig. 1:**
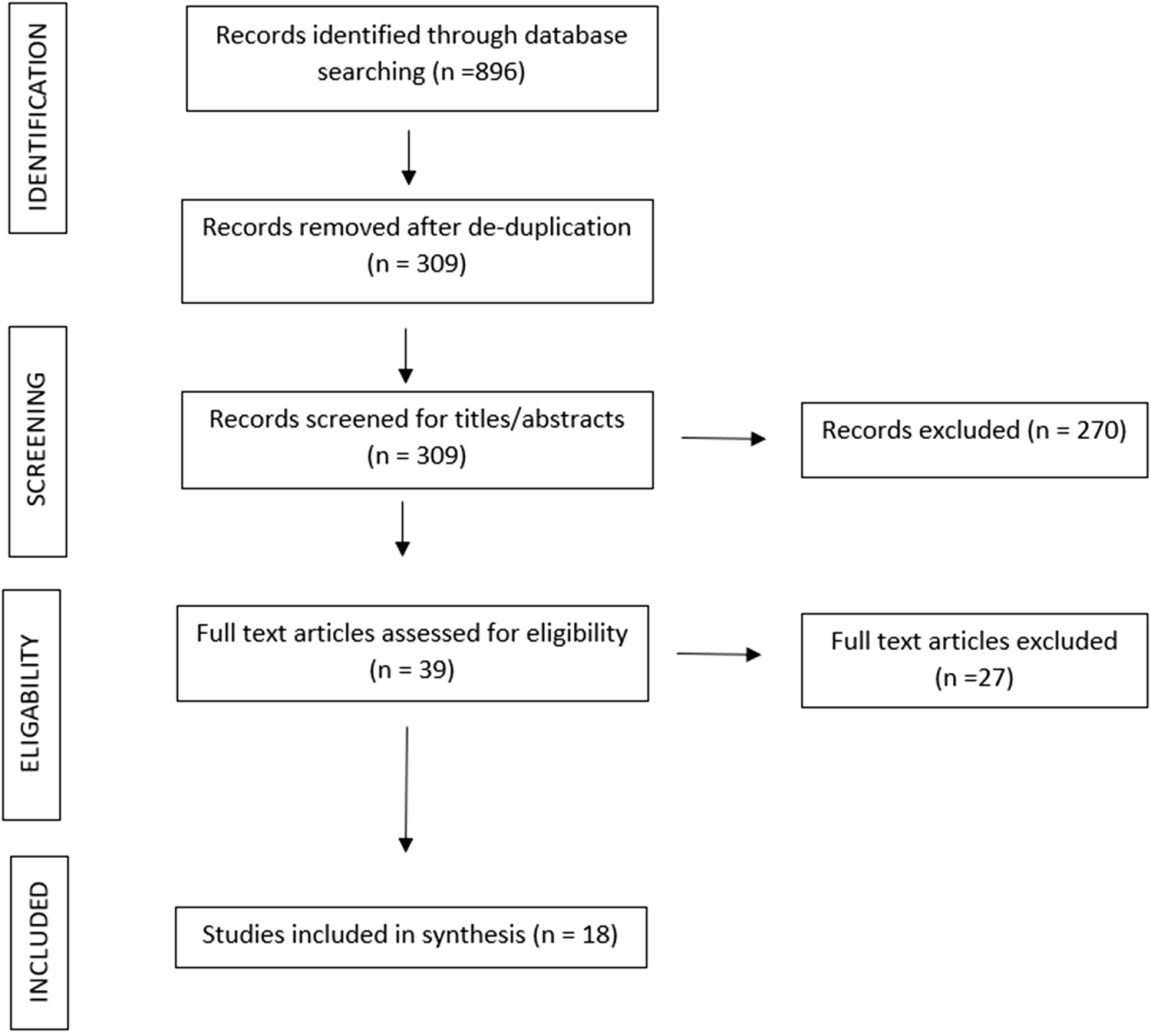
PRISMA study selection flow diagram

A total of 896 studies were identified from the database search, and upon de-duplication, 309 studies remained. A total of 270 papers were removed upon evaluation of the title and abstract, and then 21 articles were removed through screening of the full text, leaving a total of 18 studies to include in the final synthesis. From the included studies, 17 were peer-reviewed, full text articles. One of the identified studies [35] was only available as an abstract, but the poster presentation was made available with sufficient data included and so was retained in this review. This is presented as per the PRISMA reporting guidelines [33] in Fig. 1.

### Characteristics of included studies

The characteristics and information from the 18 studies can be seen in Table 1. The included studies were published between 2004 and 2023 and included a total of 420 people, 385 patients (40 under the age of 18, 345 over the age of 18) and 25 carers [35, 44]. There were also 10 health professional interviews which views were not considered in this review [44]. The studies included a variety of cancer diagnoses. The solid tumours included lung, gynaecological, breast, colorectal, upper gastrointestinal, pancreatic, glioblastoma, sarcoma, and non-solid included myeloma, lymphoma and leukaemia. Seven studies included people with advanced cancers considered to be palliative [34–40], 10 included those with curable cancers [41–50] and one study included mixed [51]. Of the included studies, 10 received exercise or physical activity intervention [37–39, 41–43, 45, 46, 49, 50]. Eight studies did not have an exercise or PA intervention, focussing solely on people’s perceptions on exercising or partaking in PA during treatment [34–36, 40, 40, 44, 48, 51].

**Table 1:** Summary data extraction table for included studies

Author	Country	Data collection method	Sampling approach	Participant characteristics	Curative or palliative	Tumour group	Quality appraisal (JBI)
Adamsen et al. 2009 [41]	Denmark	Semi-structured interviews	Not specified	22 patients. Median age 28 (range 18–40)	Curative	Breast, colon, testis, cervix, sarcoma, UGI, haematological, AML/ALL	5
Adamsen et al. 2011 [42]	Denmark	Semi-structured interviews	Purposive (embedded within an intervention)	15 patients. No age given	Curative	Lung	7
Adamsen et al. 2017 [50]	Denmark	Semi-structured interviews	Purposive	33 patients. Median age 49 (range 31–73)	Curative	Breast and colon post-surgery	6
Bland et al. 2022 [34]	Australia	Semi-structured interviews	Not specified	20 patients. Mean age 61 years (SD 13)	Palliative	Metastatic or locally advanced cancer	9
Chang et al. 2020 [35]	Taiwan	Semi-structured interviews	Purposive	24 patients. Mean age 60.3 years (range 49–78)	Palliative	Metastatic lung cancer	5
Coon & Coleman 2004 [43]	USA	Semi-structured interviews	Purposive	21 patients. Median age 52 (range 36–70)	Curative	Myeloma	10
Depenbusch et al. 2022 [36]	Germany	Focus group	Not specified	44 patients. Mean age 52 (SD 9)	Palliative	Metastatic breast cancer	5
Dennett, Harding and Reed 2020 [44]	Australia	Semi-structured interviews	Purposive	9 patients. Mean age 67.1 (SD 8.2)	Curative	Mixed	10
Edbrooke et al. 2020 [37]	Australia	Semi-structured interviews	Non-random sampling	25 participants. Mean age 66.5 (SD 12.5)	Palliative	Small cell carcinoma (stages 3 and stage 4)	5
Gotte et al. 2014 [38]	Germany	Semi-structured interviews	Not specified	40 patients. Mean age 13 (SD 4.1)	Palliative	ALL, AML, myeloma, lymphoma, sarcoma	5
Halkett et al. 2020 [39]	Australia	Semi-structured interviews	Not specified	19 patients and 15 carers. Mean age 53 (SD 11.76)	Palliative	Glioblastoma	7
Hatlevoll et al. 2021 [45]	Norway	Semi-structured interviews	not specified	15 patients. Median age 65 (range 43–80)	Curative	Colorectal	5
Mikkelsen et al. 2019 [40]	Denmark	Semi-structured interviews	Purposive	23 patients. Median age 72 (range 65–85)	Palliative	Lung, pancreatic, biliary	6
Mikkelsen et al. 2021 [46]	Denmark	Semi-structured interviews	Not specified	18 patients. Median age 71 (IQR 68–76)	Curative	Non-small cell cancer, pancreatic and biliary tract cancer	5
Polen-De et al. 2021 [47]	USA	Semi-structured interviews	Not specified	15 patients. Mean age 64.3 (no SD)	Curative	Ovarian	4
Romero-Elias et al. 2020 [48]	Spain	Semi-structured interviews	Not specified	10 patients. Mean age 58.5 (range 35–75). 10 relatives and 10 health professionals	Curative	Colorectal	5
Smith et al. 2022 [51]	UK	Focus group	Not specified	18 patients. Mean age 60.5 years (Range 32–80)	Curative and palliative	Prostate cancer, non Hodgkin lymphoma, breast, Hodgkin lymphoma	7
Yu et al. 2020 [49]	Korea	Semi-structured interviews	Purposive	6 patients. Medium or mean age not reported, but age range of 30–60 years reported	Curative	Haematological—stem cell transplant	6

The studies were conducted across 10 countries: Korea (*N* = 1), Denmark (*N* = 5), Australia (*N* = 4), the USA (*N* = 2), Germany (*N* = 2), Spain (N = 1), Taiwan (*N* = 1), Norway (*N* = 1) and the UK (*N* = 1). Two of the included studies collected data through focus groups [36, 49], and the remaining studies used semi-structured interviews.

### Quality appraisal

The JBI quality appraisal checklist was used to assess quality of all included studies. Study quality overall was good with 17 of the included studies presenting with a score of 5 or more out of 10. Table 2 demonstrates this in more detail.

**Table 2:** JBI quality appraisal

JBI Critical Appraisal Tool element	Is there congruity between the stated philosophical perspective and the research methodology?	Is there congruity between the research methodology and the research question or objectives?	Is there congruity between the research methodology and the methods used to collect data?	Is there congruity between the research methodology and the representation and analysis of data?	Is there congruity between the research methodology and the interpretation of results?	Is there a statement locating the researcher culturally or theoretically?	Is the influence of the researcher on the research, and vice- versa, addressed?	Are participants, and their voices, adequately represented?	Is the research ethical according to current criteria / evidence of ethical approval?	Do the conclusions drawn in the research report flow from the analysis, or interpretation, of the data?	Overall score of quality:
Adamsen et al. 2009 [41]	Yes	Yes	Yes	Unclear	Yes	No	No	Unclear	No	Yes	5
Adamsen et al. 2011 [42]	Yes	Yes	Yes	Yes	Yes	No	No	Yes	No	Yes	7
Adamsen et al. 2017 [50]	Yes	Yes	Yes	Unclear	Yes	No	No	Unclear	Yes	Yes	6
Bland et al. 2022 [34]	Yes	Yes	Yes	Yes	Yes	Yes	Yes	Unclear	Yes	Yes	9
Chang et al. 2020 [35]	Unclear	Yes	Yes	Yes	Yes	No	No	Unclear	Yes	Unclear	5
Coon & Coleman 2004 [43]	Yes	Yes	Yes	Yes	Yes	Yes	Yes	Yes	Yes	Yes	10
Dennett et al. 2020 [36]	Yes	Yes	Yes	Yes	Yes	Yes	Yes	Yes	Yes	Yes	10
Depenbusch et al. 2020 [36]	Yes	Yes	Yes	Yes	No	No	No	No	Unclear	Yes	5
Edbrooke et al. 2019 [37]	Unclear	Unclear	Yes	Yes	Yes	No	No	Yes	Yes	Unclear	5
Gotte et al. 2014 [38]	Unclear	Yes	Yes	Unclear	Yes	No	No	Unclear	Yes	Yes	5
Halkett et al. 2020 [39]	Yes	Yes	Yes	Yes	Yes	No	No	Unclear	Yes	Yes	7
Hatlevoll et al. 2021 [45]	Unclear	Yes	Yes	Unclear	Yes	No	No	Unclear	Yes	Yes	5
Mikkelsen et al. 2019 [40]	Unclear	Yes	Yes	Yes	Yes	No	No	Unclear	Yes	Yes	6
Mikkelsen et al. 2022 [46]	Unclear	Yes	Yes	Unclear	Yes	No	No	Unclear	Yes	Yes	5
Polen-De et al. 2021 [47]	Unclear	Unclear	Unclear	Yes	Yes	No	No	Yes	Yes	Unclear	4
Romero-Elias et al. 2020 [48]	Unclear	Yes	Yes	Unclear	Yes	No	No	Unclear	Yes	Yes	5
Smith et al. 2022 [51]	Yes	Yes	Yes	Unclear	Yes	No	Yes	Unclear	Yes	Yes	7
Yu et al. 2020 [49]	Unclear	Yes	Yes	Yes	Yes	No	No	Yes	No	Yes	6

All studies had good congruence between the research methodology and the research objectives, and most ensured patient voice was adequately represented. Few provided statements on locating the researcher culturally or theoretically, and few discussed researcher reflexivity. The individual study scores for the quality appraisal may be seen in Table 1.

### Synthesis

**Fig. 2:**
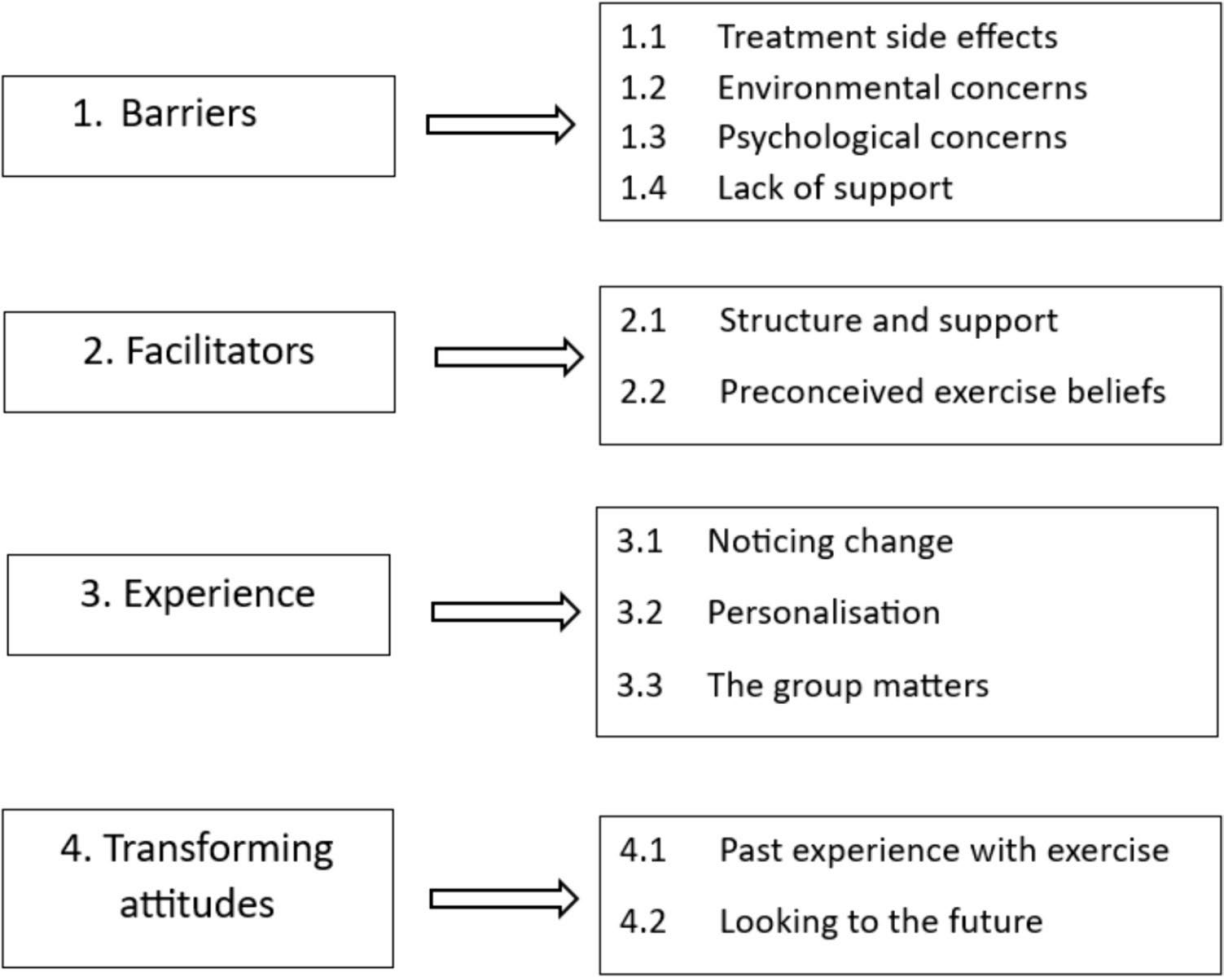
Summary of the combined themes and subthemes from the data synthesis

Four main themes emerged from the qualitative synthesis: (1) Barriers, (2) Facilitators, (3) Experience of PA and exercise and (4) Transforming attitudes. The themes and sub-themes are presented in Fig. 2.

### Barriers

This theme encompasses the difficulties and barriers people undergoing cancer treatment perceive and face when it comes to physical activity and exercise. It has been categorised into four sub-themes: treatment side effects, environmental concerns, psychological concerns and lack of support.

### Treatment side effects

Many participants expressed that side effects from oncological treatment were significant barriers for participating in physical activity or exercise with fatigue often being highlighted as the most severe.

“Fatigue is the main barrier to do exercise, now I'm on chemotherapy and I feel tired. I used to walk four or five km a day … Now, really, the chemotherapy exhausts me … and I don't have any spirit to do physical activity” [48].

“After the treatment obviously that [exercise] dropped off down a cliff edge. I just couldn’t do it whatsoever after a couple of weeks of treatment. I was quite lethargic; everything was hard to do” [51].

### Environmental concerns

Many participants expressed environmental concerns as a barrier to exercise including lack of suitable environments to exercise in, the weather and financial burden.

“And having a one-on-one, like having a personal trainer, I would say financially because I don’t work, and I don’t have a pension. So, you know, it’s just money out of your own savings, really” [34].

“I don't think that sport centres are prepared for cancer patients … and I'm not going to go centre by centre asking if they have activities adapted for me, because I don't want to explain my case to everyone” [48].

### Psychological concerns

Psychological barriers were also expressed throughout the studies, including fear of making symptoms or disease worse, fear of losing weight due to exercise, low self-esteem and motivation.

“I needed a cane to go out walking because of spine metastasis and leg weakness, so I still thought, could I go out? I felt very stressful when the stranger looks at me” [35].

“Because I found out that if I burned 1, 2, 3, 4, 5, 600 cal every hour, then I would not be able to catch up (with that). Meaning, I would experience a bigger weight loss by exercising. I simply could not afford it. So, I stopped” [40].

### Lack of support

Participants also reported that a lack of support, education and awareness from healthcare professions left them feeling unconfident in their ability or unsure of where to start with physical activity and exercise.

“I used to bring that up all the time [exercise], with them [consultant]. Every week I brought it up. You are kind of just left to guide your way through your own recovery” [51].

“I’ve just taken it for myself to go and YouTube and punched in how to strengthen muscles after chemotherapy…it would be nice if somebody at the hospital had that expertise” [44].

### Facilitators

This theme encompasses the perceptions of the participants on facilitators to them participating in physical activity or exercise and has been categorised into two sub-themes: structure and support and preconceived exercise beliefs.

#### Structure and support

Many participants expressed the importance of support from healthcare professionals and structure to physical activity and exercise as being key in partaking in more exercise and physical activity.

“The encouragement I received from exercise therapists when I was having a hard time doing the exercise alone helped me to carry on. Encouraging words (motivational talks) might not have been that important, but really helped me to stay determined” [49].

“I felt safe as it was all placed at the same location. It was my impression that they all knew what was going on across the different departments. It provided security that the people around me all knew my story” [46].

#### Preconceived exercise and physical activity beliefs

Positive preconceived beliefs about physical activity were expressed in many studies, with many participants having a positive outlook on how physical activity may help them during their cancer treatment.

“I felt physically weak. The more strength I get from training the more resistance I will have to use against my illness” [41].

“Exercise is something that I can do. I cannot cure myself, but I CAN do this (voice is cracking). I think it’s so important to have that counterplay. To feel physically strong, because that will also affect your mentality. And if you are physically strong, then you might tolerate the next treatment better” [46].

### Experience of PA/exercise

This theme describes people’s experience with exercise and can be categorised into three sub-themes: noticing change, personalisation and the group matters.

#### Noticing change

Many participants reported that noticing change in symptoms, strength and psychological health was a positive outcome of exercise and increased motivation further.

“Really just made me feel better, that I could do it, and that I’m glad that as I got better, you know, my body got more limber and your coordination gets better every morning as you stretch and do those exercises” [43].

“You feel better after you have done the exercise….I feel that it helps you get to be able to do more things, lead a more normal life. It makes you feel a little bit healthier, um It takes away the feeling of, um, um, sickness, you know, you do not feel it to such an extent” [37].

#### Personalisation

A few participants highlighted that a personalised exercise programme and support was important to them during exercise or physical activity advice.

“It was all tailored and the trainer was monitoring me. When she could see that I was taxed she eased off a little” [39]

#### The group matters

Almost all participants in all studies discussed that experiencing in a group setting was a positive experience for them.

“Talking about the cancer wasn’t easy for me at first. So, when a teammate approached me and said: ‘Hi, my name is x, I have this cancer. Where do you have cancer?,’ I was shocked. But I was positively surprised about all these people. Even though we were all sick and were so different in many other ways, we had so much fun together” [46].

“Training in a group is much better because you know that someone is keeping an eye out whether you come or not. It’s a little more difficult when you have to do it on your own” [42].

### Transforming attitudes

This theme highlights participants’ previous experience with physical activity or exercise and how this may have changed when assessing what their physical activity or exercise experience may look like in future. It has been categorised into two sub-themes: past experience with exercise and looking to the future.

#### Past experience with exercise

One study included young-athletes diagnosed with cancer, so these participants often had extensive experience exercising pre-diagnosis.

“I have always liked sports so I have always been able to maintain a well-trained body; not a pumped up look but well-trained” [41].

However, the majority of participants had minimal experience with exercise, often putting this down to stressful jobs or busy home lives.

“It was never a priority… I would put myself as a zero for physical activity before I was diagnosed” [47].

#### Looking forwards

Many participants stressed a desire to increase their physical activity in future, especially those studies that were focussing on patient experience post an exercise intervention as part of their study.

“I'm going to continue going to the gym with the exercise physiologist” [39].

“I want to continue being physically active at a training club for elderly people” [41].

## Discussion

This review has explored people’s attitudes and experiences towards exercise and physical activity when undergoing oncological treatment and identified four main themes: barriers, facilitators, experience and transforming attitudes. The review included a wide range of tumour groups, age ranges and cancer stage from a variety of countries to ensure experiences and attitudes were represented a broad range of the population. This is essential to better understand people’s experience when advising people undergoing any type of oncological treatment to exercise or when implementing structured exercise or physical activity intervention in both the clinical setting and in future research.

From the included studies, there was an overall positive perception of exercise or physical activity, and people could see potential benefits of how it could help them during their cancer treatment. Most included participants had little to no previous experience with regular physical activity or exercise. Many reported busy careers and family life being a barrier to this which is a common barrier to exercise in the general population [52]. These points both provide a key starting point when discussing the results of this review: that people have a positive attitude towards exercise and its potential benefits during cancer treatment; however, many have not previously taken part in exercise. This is encouraging, as it shows willingness to partake in exercise or physical activity, and this discussion aims to highlight barriers and facilitators to do so.

When discussing exercise, participants in almost all included studies reported fatigue being a significant barrier. A systematic review and meta-analysis reported that fatigue in people with cancer was present in 43–62% of people internationally [53]. This is supported by the most recent UK national cancer patient experience survey (*N* = 20,000) that reported 68% of participants felt exhausted or fatigued [54]. It is widely documented that physical activity and exercise reduce self-reported cancer-related fatigue [55]; however, a qualitative review found that 58% of people living with cancer did not feel well informed about fatigue, and 41% had never been asked about fatigue by their treating physician [56]. This suggests lack of understanding about fatigue by the person undergoing treatment as well as lack of confidence or understanding of how to discuss and advise on cancer-related fatigue by healthcare professionals (HCPs). This may suggest that educational resources for both people undergoing oncological treatment and HCPs are needed to reduce fatigue as a barrier to physical activity/exercise.

This leads onto the next barrier to physical activity and exercise reported in many of the included articles, lack of support from HCPs. When interviewing healthcare professionals (HCPs), many report low awareness and lack of confidence in knowing what to advocate for when discussing rehabilitation and exercise with people with cancer [44]. Furthermore, one study found that 33–50% of HCPs have poor self-knowledge of when, how and which cancer survivors to refer to exercise programmes and how to counsel based on exercise guidelines [57]. Participants in this review reported not knowing which form of exercise was safe, what they could or could not do and that lack of support or guidance from HCPs was a significant barrier. The need for HCP support and guidance when undertaking exercise is also reported in qualitative studies from people suffering from other conditions such as Parkinsons, stroke and people undergoing kidney transplant [58–60]. There is significant work underway to improve HCP understanding of prescribing exercise and physical activity, such as the ‘Moving Medicine’ programme in the UK [61] and the ‘Exercise is Medicine in Oncology’ clinician engagement work by international experts in exercise for oncology [62]. This demonstrates awareness that intervention is needed to improve the way HCPs recommend exercise and physical activity but much more needs to be done to improve HCP knowledge to reduce this barrier to physical activity and exercise.

Another significant barrier reported in many of the included studies was being unable to access community exercise or physical activity interventions locally to them: that travel to tertiary centres was often expensive and took a significant amount of time that participants were unwilling to do. Some also reported worry that local centres would not understand their cancer diagnosis and be unable to support them through this complex time. The ‘Right to Rehab’ report published by 24 charities, trade unions and professional bodies highlighted the huge disparity for people to access community rehabilitation throughout the UK [63]. Within the report, they discuss the lack of community rehabilitation for people with cancer specifically and that there is not equal access across the UK. Furthermore, a recent survey completed by the Chartered Standard of Physiotherapists found that many hospital and community rehabilitation spaces were converted to discharge lounges, storage spaces and overspill wards during the COVID-19 pandemic and have not been returned to use for rehabilitation, with 50% of Physiotherapists seeing fewer patients per week [64]. This demonstrates that there is a huge amount of work to be done to improve access to community rehabilitation before this reported barrier can be reduced.

Some participants reported body-image concerns as a barrier to participating in physical activity and exercise, especially the study on the views of young athletes with cancer. It is well documented that many people with cancer can struggle with body image issues both during and after treatment [65]. In people with breast or prostate cancer, exercise has been shown to positively impact body image and personal identity [66], and the young athletes in the included article reported once they started to see positive changes to their body and perceived body image when engaging with exercise and physical activity, this became a facilitator to continue. This leads onto the repeatedly reported sub-themes within patient experience: noticing change.

After exercise, many participants reported experiencing improvements in treatment side effects such as nausea, pain and fatigue which was a key motivator to continue participating in exercise and physical activity during cancer treatment. As stated in the introduction, it is widely published that exercise and physical activity can improve side effects of oncological treatment [13, 14]. This suggests that informing and educating people undergoing oncological treatment of the benefits exercise can have on their side effects is key. Furthermore, many participants reported that during exercise, being supported to set personalised, meaningful goals was a significant facilitator and helped maintain motivation; further affirming the need for HCP support is essential. Participants in two of the included studies in this review reported the provision of a pedometer or heart rate monitor useful in maintaining motivation when exercising [44, 49]. A systematic review in cancer survivors found the use of pedometers to monitor progress increased the total amount of physical activity as well as increasing moderate-to-vigorous intensity exercise and improving physical function [67]. Overall, noticing positive change in symptoms during treatment was a facilitator to physical activity and exercise with some reporting personalisation of goal setting and provision of useful ways to monitor this change maintains motivation.

Another motivating factor repeatedly reported throughout many studies was being able to participate in exercise in a group setting. People reported it not just being a motivator to continue with exercise, but also a psychological support when dealing with their diagnosis. This group setting providing social support and motivation is widely documented in people with other conditions such as Parkinson’s disease, post organ transplant and elderly people who have had multiple falls [59, 68, 69]. Some studies reported that participants liked having the option of following a structured programme at home, showing that choice is important. Overall, this highlights people find a group setting motivational, but the option to do exercise at home is also important and so requires a personalised approach.

To conclude, people undergoing cancer treatment have a positive perception of exercise benefits and, once positive change is noticed, often in a local group exercise setting, motivation to continue exercise or physical activity increases. However, to begin exercising, support from HCPs in prescribing exercise and discussing the positive effect this could have on reducing symptom burden is essential, but according to wider literature, confidence and knowledge to do so can often be low in HCPs.

## Implications for future research

The barrier of lack of support from HCPs was reported throughout. Future research should focus on improving HCP knowledge and confidence when discussing the benefits of exercise and prescribing exercise or physical activity to those undergoing oncological treatment. Furthermore, future research studies implementing an exercise intervention should utilise this evidence synthesis to minimise perceived barriers which will ensure maximal compliance and improve patient experience with the given intervention.

## Limitations

When assessing study quality using the JBI appraisal tool, 9 of the 18 studies scored 5/10 or less. Scores were often reduced on the quality tool through unclear reporting instead of low research rigour. Most studies that provided an exercise intervention used the same researcher for both the exercise and the qualitative follow-up with no reflexive account as to how this may have influenced the outcome of the research. These studies were not excluded, as it was deemed that the experience reported by the included participants was important to the review findings. This review included qualitative studies in which the participants had not been provided with an exercise or PA intervention prior to the study. This could be seen as a limitation, as the participants had not experienced a designed exercise or PA intervention. However, as many specifically designed exercise intervention for those with cancer is variable and often not available at all, it was decided including these studies could provide greater insight into barriers and experience with exercising or engaging with physical activity independently throughout treatment. A further limitation is that all studies, apart from two (one from Taiwan and another from Korea), were from western countries. This may not represent international experiences of people undergoing oncological treatment and therefore would warrant further information. Finally, out of the 18 included studies, only two were specific to a paediatric population. When utilising the qualitative data to design and/or improve exercise services or PA recommendations for children undergoing oncological treatment, results should be interpreted with caution as the majority of studies were exploring adult perceptions, experiences and barriers, not children.

## Conclusions

Despite published reported adherence to exercise and physical activity guidelines being low in people with cancer, this review found that people have a positive perception of exercise or physical activity and could see why this may benefit them during cancer treatment. This review discusses those barriers and facilitators that may support people in increasing their exercise and physical activity adherence. A key facilitator was that support from HCPs is essential, but wider research shows that many HCPs may lack confidence in discussing benefits of exercise and prescribing exercise to those undergoing oncological treatment. This highlights an opportunity to reduce this barrier to physical activity and exercise by improving HCP knowledge and confidence when discussing and prescribing exercise and should be a key focus on future research interventions. Furthermore, the participants in this review report that personalised exercise in a local, group setting was preferred as this provided both motivation, positive change in both physical and psychological symptoms as well as social support. When designing future exercise interventions for those undergoing treatment, these experiences and facilitators should be taken into consideration to improve patient experience and maximise adherence.

## Data Availability

All data generated and/or analysed during this review are included in the published article and its supplementary files.

## Appendix 1 Search strategy for Embase and Medline

Database: Embase < 1974 to 2022 September 29 > , Ovid MEDLINE(R) ALL < 1946 to September 29, 2022 > 

Search Strategy
